# The effect of maternal poliovirus antibodies on the immune responses of infants to poliovirus vaccines

**DOI:** 10.1186/s12879-020-05348-1

**Published:** 2020-08-31

**Authors:** Siyue Jia, Rong Tang, Guifan Li, Yuemei Hu, Qi Liang

**Affiliations:** 1 Jiangsu Provincial Center for Disease Control and Prevention, NO. 172 Jiangsu Rd., Gulou District, Nanjing, 210009 Jiangsu Province China; 2 Beijing Minhai Biotechnology Co. Ltd., Beijing, China

**Keywords:** Poliovirus vaccine, Maternal antibody, Immune response

## Abstract

**Background:**

Maternal poliovirus antibodies could provide passive immunity to the newborns from poliovirus infection during their first few months of life, but they may impair the immune responses of infants to the poliovirus vaccine as well. In our study, we pooled the data from three clinical trials of the inactivated poliovirus vaccine (IPV) based on Sabin strains to investigate the effect of maternal poliovirus antibodies on the immune responses of infants to poliovirus vaccines.

**Methods:**

There were five groups in the pooled analysis, including low-dose Sabin IPV, medium-dose Sabin IPV, high-dose Sabin IPV, control Sabin IPV, and control Salk IPV groups. We reclassified the infants in different groups according to their maternal poliovirus antibodies by two methods, the first one included maternal antibody negative (< 1:8) and maternal antibody positive (≥1:8), and the second one included maternal antibody titer < 1:8, 1:8 ~ < 1:32 and ≥ 1:32. Then, we compared the geometric mean titers (GMTs), geometric mean antibody fold increases (GMIs) and seroconversion rates of poliovirus type-specific neutralizing antibodies after vaccination among participants with different maternal poliovirus antibody levels.

**Results:**

The GMTs and GMIs of three types of poliovirus antibodies after vaccination in maternal antibody negative participants were significantly higher than those in maternal antibody positive participants. The seroconversion rates of type II and type III poliovirus antibodies in maternal antibody positive participants were significantly lower than those in maternal antibody negative participants. Among participants with maternal antibody titer < 1:8, 1:8 ~ < 1:32 and ≥ 1:32, the GMTs and GMIs of three types of poliovirus antibodies after vaccination showed a tendency to decline with the increasing of maternal antibody levels. The seroconversion rates of three types of poliovirus antibodies in participants with maternal antibody titer ≥1:32 were significantly lower than those in participants with maternal antibody titer < 1:8 and 1:8 ~ < 1:32.

**Conclusions:**

Maternal poliovirus antibodies interfered with the immune responses of infants to poliovirus vaccines, and a high level of maternal antibodies exhibited a greater dampening effect.

**Trial registration:**

ClinicalTrials.govNCT04264598 February 11, 2020; ClinicalTrials.govNCT04264546 February 11, 2020; ClinicalTrials.govNCT03902054 April 3, 2019. Retrospectively registered.

## Background

Polio was once a disease feared worldwide, which has been reduced by 99% due to the implementation of the Global Polio Eradication Initiative [1]. On October 17, 2019, World Health Organization (WHO) declared that wild poliovirus type II and type III have been eradicated worldwide [2]. While polio is a distant memory in most of the world, the disease still exists in some places and mainly affects children under five. Oral poliovirus vaccine (OPV) and inactivated poliovirus vaccine (IPV) are currently used to prevent polio across the world. OPV is one of the safest vaccines ever developed, which can be given to sick children and newborns [3]. On extremely rare occasions, the attenuated virus in OPV can mutate and regain virulence [4]. Some countries have switched from OPV to IPV to decrease the risk of emerging virulent poliovirus revertants. Since 2000, the United States has replaced OPV with IPV to eliminate the risk of vaccine-derived polio [5], while OPV is still used in some parts of the world, especially in developing countries, because of its cheapness. At present, there are two IPVs on the market, namely Sabin IPV and Salk IPV. The production and quality control of Salk IPV require at least a biosafety level 3 containment facility, while those of IPV based on Sabin strains will have a lower biosafety risk, and increase the availability and affordability of IPV in the low- or middle-income countries [6, 7].

Maternal antibodies are transferred to infants via the placenta during the third trimester of pregnancy and provide passive immunity to the newborns from infections during their first few months of life [8]. However, passively acquired maternal antibodies have been found to impair the immune responses of infants to measles [9], hepatitis A [10] and hepatitis B [11] vaccines. The results of a meta-analysis showed that maternal antibody concentrations and infant age at first vaccination both influence infant vaccine responses, and these effects are seen for almost all vaccines in the global immunization programs and influence the immune responses for some vaccines even at the age of 2 years [12]. Several studies have shown that maternal antibodies may impair the immune responses of infants to Salk IPV as well [13–16].

As WHO recommends Sabin IPV in the development of affordable next generation IPVs, and more countries look to move towards IPV schedules, it is critical to determine the effect of maternal antibodies on the response to Sabin IPV so as to optimize the effectiveness of it in infants. We have conducted three clinical trials to evaluate the safety and immunogenicity of an IPV based on Sabin strains. In this study, we pooled the data from these three clinical trials to investigate the effect of maternal poliovirus antibodies on the immune responses of infants to poliovirus vaccines, including the investigational Sabin IPV with different antigen contents, licensed Sabin IPV and Salk IPV.

## Methods

### Study design

Between August, 2017, and December, 2018, we conducted three clinical trials to evaluate the safety and immunogenicity of an IPV based on Sabin strains at two sites in Lianshui County and Dafeng District, Jiangsu Province, China. In the phase I trial (ClinicalTrials.gov: NCT04264598), 60 infants aged 2 months were randomly assigned in a 1:1:1 ratio to receive the medium-dose Sabin IPV, control Sabin IPV or control Salk IPV. In the phase Ib trial (ClinicalTrials.gov: NCT04264546), 20 infants aged 2 months were administered with the high-dose Sabin IPV, with no control group. In the phase II trial (ClinicalTrials.gov: NCT03902054), 600 infants aged 2 months were randomly assigned equally to receive the low-dose Sabin IPV, medium-dose Sabin IPV, high-dose Sabin IPV, control Sabin IPV or control Salk IPV. All infants were administered with 3 doses of IPV at 2, 3, and 4 months of age. Blood samples were collected immediately before the first dose and 30 days after the third dose for serum poliovirus type-specific neutralizing antibody detection, which was performed by the Chinese National Institute for Food and Drug Control, according to the method recommended by the WHO [17]. All Infants who completed the three-dose vaccination and had pre- and post-vaccination antibody detection results were included in the pooled analysis. According to the poliovirus neutralizing antibodies before vaccination, which could be considered as maternal antibodies, the infants in each group were reclassified. We used two classification methods. The first method was to divide the infants in each group into two subgroups, namely maternal antibody negative (< 1:8) and maternal antibody positive (≥1:8). The second method was to divide the infants in each group into three subgroups, namely maternal antibody titer < 1:8, 1:8 ~ < 1:32 and ≥ 1:32. After that, we compared the geometric mean titers (GMTs), geometric mean antibody fold increases (GMIs) and seroconversion rates of poliovirus type-specific neutralizing antibodies after vaccination among participants with different maternal poliovirus antibody levels, to investigate the effect of maternal poliovirus antibodies on the immune responses of infants to poliovirus vaccines.

### Participants

The population of Lianshui County is about 850,000, and that of Dafeng District is about 700,000. The total number of infants aged 0–12 months at the two sites is about 18,000, which the trial participants were recruited from. The inclusion and exclusion criteria for infant participants in phase I, Ib and II trials were consistent. Eligible participants were healthy infants aged between 60 and 90 days, with no history and contraindication of poliovirus vaccination. Exclusion criteria were a history of polio, premature or low birth weight, congenital malformation or developmental disorders, a history of seizure or mental diseases, immunodeficiency or receiving immunosuppressive therapy, receipt of any blood products in the past 3 months, receipt of any live attenuated vaccines in the past 14 days, receipt of any subunit or inactivated vaccines in the past 7 days, receipt of any other research drugs, or other factors that were not suitable for clinical trials according to the judgment of researchers.

### Ethics statement

The protocols and informed consent documents for the three clinical trials in the pooled analysis were approved by the Institutional Review Board of the Jiangsu Provincial Center of Disease Control and Prevention. Written informed consent was obtained for the guardians of all participants. The clinical trials were done in accordance with the Declaration of Helsinki and Good Clinical Practice guidelines.

### Vaccines

The investigational Sabin IPV (low-dose, medium-dose and high-dose) were all developed by Beijing Minhai Biotechnology Co., Ltd. The control Sabin IPV and control Salk IPV were manufactured by Institute of Medical Biology Chinese Academy of Medical Sciences and Sanofi Pasteur S. A, respectively. The D antigen units (DU) of type I, type II and type III polioviruses in the investigational and control vaccines were as follows: low-dose Sabin IPV (10 DU, 30 DU and 30 DU), medium-dose Sabin IPV (15 DU, 45 DU and 45 DU), high-dose Sabin IPV (22 DU, 65 DU and 65 DU), control Sabin IPV (30 DU, 32 DU and 45 DU), control Salk IPV (40 DU, 8 DU and 32 DU). All vaccines were in liquid form, 0.5 ml per dose.

### Statistical analysis

Samples with antibody titers below the detection limit (1:8) were given an arbitrary value of 1:4 for calculations. Antibody titers were log-transformed in order to calculate the GMTs and GMIs. We used one-way ANOVA or Wilcoxon rank-sum test to compare the GMTs and GMIs of poliovirus type-specific neutralizing antibodies after vaccination among participants with different maternal poliovirus antibody levels. The seroconversion rates (defined as pre-vaccination titer less than 1:8 and post-vaccination titer 1:8 or more, or pre-vaccination titer 1:8 or more and at least four-fold increase post-vaccination) among participants with different maternal poliovirus antibody levels were compared using χ^2^ test or Fisher’s exact test. When a significant difference was found, further pairwise comparisons were performed and Bonferoni-adjusted *P* values were calculated. All reported *P* values were 2 sided, and values less than 0.05 were regarded as statistically significant. Statistical analyses were performed by using SAS 9.3 software (SAS Institute, Inc., Cary, NC, USA).

## Results

### Study participants

**Fig. 1:**
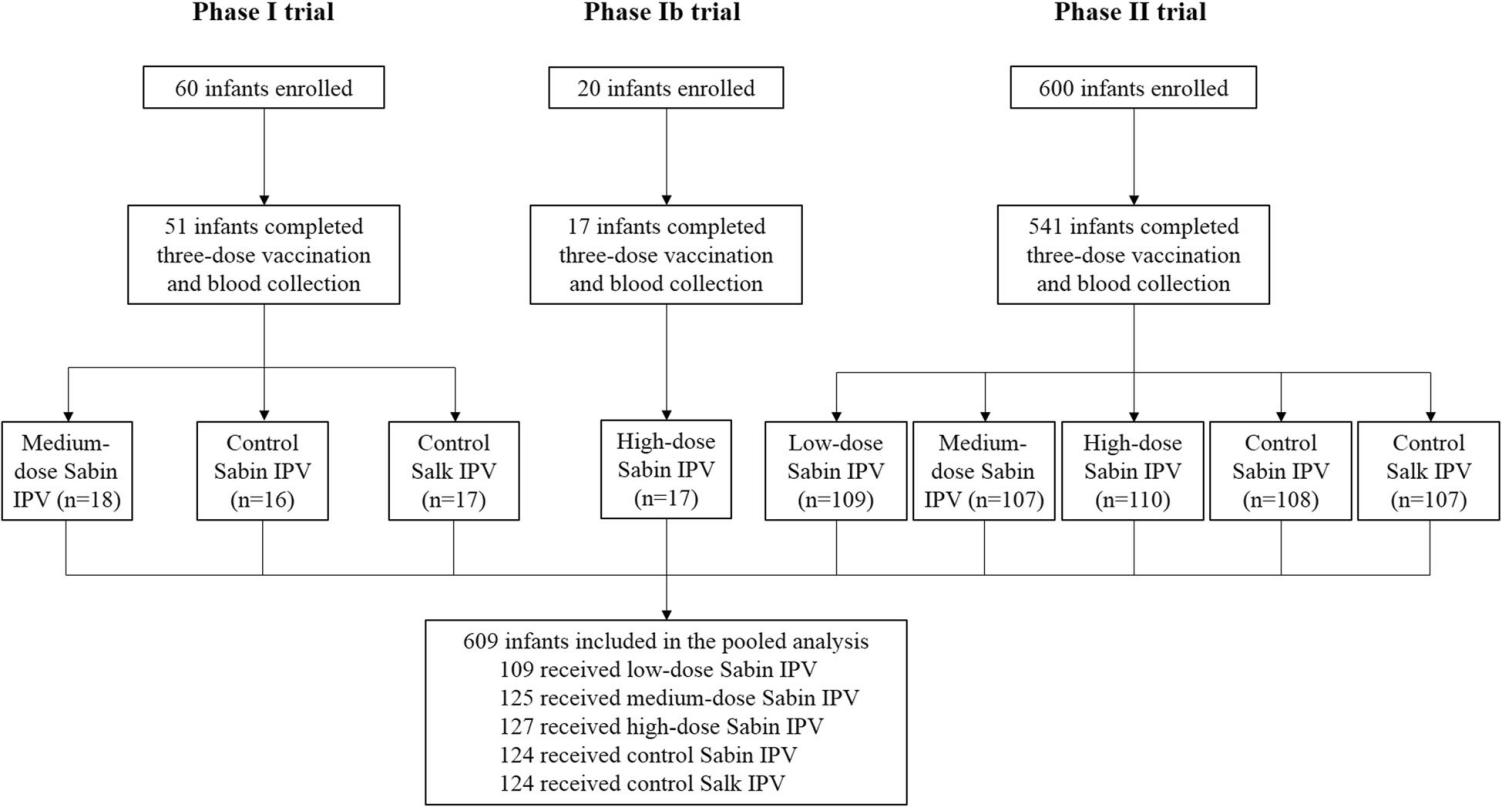
Pooled analysis profile

Figure 1 shows the pooled analysis profile. Six hundred nine infants who completed three-dose vaccination and blood collection were included in the pooled analysis. Of them, 109 received low-dose Sabin IPV, 125 received medium-dose Sabin IPV, 127 received high-dose Sabin IPV, 124 received control Sabin IPV, and 124 received control Salk IPV. Baseline demographic characteristics of the participants were similar among the five vaccine groups (Table 1). Table 2 shows the maternal poliovirus type-specific antibody levels of the participants. The positive rates of maternal antibodies in low-dose Sabin IPV group, medium-dose Sabin IPV group, high-dose Sabin IPV group, control Sabin IPV group and control Salk IPV group ranged from 70.87 to 78.40% for type I poliovirus, 34.40 to 44.95% for type II poliovirus, 16.54 to 26.61% for type III poliovirus. The GMTs of maternal antibodies in the five vaccine groups ranged from 17.57 to 21.21 for type I poliovirus, 6.44 to 8.39 for type II poliovirus, 5.33 to 6.41 for type III poliovirus. The majority of participants in the five vaccine groups had a maternal antibody titer of 1:8 ~ < 1:32 or ≥ 1:32 for type I poliovirus, < 1:8 or 1:8 ~ < 1:32 for type II and type III polioviruses.

**Table 1:** Baseline characteristics of the study participants

Characteristics	Group 1	Group 2	Group 3	Group 4	Group 5
N	109	125	127	124	124
Age—days^a^	72.18 ± 8.93	73.00 ± 8.81	72.59 ± 8.81	72.12 ± 8.09	73.83 ± 7.89
Sex—no. (%)					
Male	49 (44.95)	68 (54.40)	65 (51.18)	61 (49.19)	63 (50.81)
Female	60 (55.05)	57 (45.60)	62 (48.82)	63 (50.81)	61 (49.19)

Group 1: low-dose Sabin IPV group. Group 2: medium-dose Sabin IPV group. Group 3: high-dose Sabin IPV group. Group 4: control Sabin IPV group. Group 5: control Salk IPV group

^a^Plus–minus values are means ± SD

**Table 2:** Maternal poliovirus type-specific antibody levels of the study participants

Group	N	Type	Positive participants	Positive rate (%)	GMT	Participants with different titers (1:)		
						< 8	8 ~ < 32	≥32
1	109	I	82	75.23	17.57	27	47	35
		II	49	44.95	8.39	60	33	16
		III	29	26.61	6.13	80	20	9
2	125	I	98	78.40	18.44	27	50	48
		II	43	34.40	6.73	82	30	13
		III	23	18.40	5.33	102	19	4
3	127	I	90	70.87	17.99	37	39	51
		II	46	36.22	6.69	81	35	11
		III	21	16.54	5.59	106	14	7
4	124	I	96	77.42	21.21	28	40	56
		II	47	37.90	6.44	77	42	5
		III	33	26.61	6.41	91	23	10
5	124	I	95	76.61	19.15	29	45	50
		II	46	37.10	6.92	78	36	10
		III	31	25.00	5.79	93	25	6

Group 1: low-dose Sabin IPV group. Group 2: medium-dose Sabin IPV group. Group 3: high-dose Sabin IPV group. Group 4: control Sabin IPV group. Group 5: control Salk IPV group

### Maternal antibody dichotomy

Table 3 shows the comparisons of GMTs, GMIs and seroconversion rates of poliovirus antibodies after vaccination between maternal antibody negative and positive participants. The GMTs of antibodies after vaccination in maternal antibody negative participants were significantly higher than those in maternal antibody positive participants for type I poliovirus in low-dose Sabin IPV group (*P* = 0.006), high-dose Sabin IPV group (*P* = 0.025) and control Sabin IPV group (*P* = 0.001), for type II poliovirus in low-dose Sabin IPV group (*P* = 0.002), medium-dose Sabin IPV group (*P* = 0.005), high-dose Sabin IPV group (*P* = 0.008) and control Salk IPV group (*P* < 0.001), for type III poliovirus in low-dose Sabin IPV group (*P* = 0.017), medium-dose Sabin IPV group (*P* = 0.003) and control Sabin IPV group (*P* < 0.001). The GMIs of three types of poliovirus antibodies in maternal antibody negative participants were significantly higher than those in maternal antibody positive participants in all five vaccine groups (*P* < 0.001). The seroconversion rates of maternal antibody negative participants for three types of polioviruses in the five vaccine groups were all 100%. The seroconversion rates of type I poliovirus antibodies in all five vaccine groups were comparable between maternal antibody positive and negative participants (*P* > 0.05). The seroconversion rates of antibodies in maternal antibody positive participants were significantly lower than those in maternal antibody negative participants for type II poliovirus in low-dose Sabin IPV group (*P* = 0.008), medium-dose Sabin IPV group (*P* = 0.023), control Sabin IPV group (*P* = 0.014) and control Salk IPV group (*P* < 0.001), for type III poliovirus in low-dose Sabin IPV group (*P* = 0.001), medium-dose Sabin IPV group (*P* = 0.006), high-dose Sabin IPV group (*P* = 0.004) and control Sabin IPV group (*P* = 0.001).

**Table 3:** Comparison of GMTs, GMIs and seroconversion rates of poliovirus type-specific neutralizing antibodies after vaccination between maternal antibody negative and positive participants

Group	Type	GMT (95% CI)			GMI (95% CI)			Seroconversion rate (%)		
		Maternal negative	Maternal positive	*P*	Maternal negative	Maternal positive	*P*	Maternal negative	Maternal positive	*P*
1	I	5086.27 (3909.17–6617.80)	3191.30 (2619.36–3888.12)	0.006	1271.57 (977.29–1654.45)	111.56 (81.88–152.00)	< 0.001	100.00	98.78	1.000
	II	878.35 (704.86–1094.54)	505.09 (381.51–668.70)	0.002	219.59 (176.21–273.63)	24.32 (15.51–38.11)	< 0.001	100.00	85.71	0.008
	III	1142.55 (961.97–1357.03)	619.06 (386.93–990.46)	0.017	285.64 (240.49–339.26)	31.13 (15.56–62.31)	< 0.001	100.00	82.76	0.001
2	I	4231.17 (3210.69–5576.00)	3139.05 (2554.03–3858.08)	0.084	1057.79 (802.67–1394.00)	111.75 (88.66–154.84)	< 0.001	100.00	98.98	1.000
	II	900.64 (767.91–1056.30)	550.11 (405.38–746.52)	0.005	225.16 (191.98–264.07)	30.25 (18.80–48.67)	< 0.001	100.00	90.70	0.023
	III	1036.17 (880.40–1219.50)	564.94 (372.86–855.96)	0.003	259.04 (220.10–304.87)	29.60 (12.55–69.80)	< 0.001	100.00	86.96	0.006
3	I	6187.48 (4563.25–8389.85)	4131.94 (3411.86–5003.98)	0.025	1546.87 (1140.81–2097.46)	123.79 (86.99–176.17)	< 0.001	100.00	96.67	0.556
	II	1018.74 (873.77–1187.76)	711.84 (567.01–893.67)	0.008	254.69 (218.44–296.94)	43.10 (29.34–63.29)	< 0.001	100.00	95.65	0.129
	III	1207.23 (1025.79–1420.76)	835.86 (475.52–1469.29)	0.206	301.81 (256.45–355.19)	27.66 (10.49–72.96)	< 0.001	100.00	85.71	0.004
4	I	5830.71 (4431.70–7671.36)	3051.49 (2523.95–3689.29)	0.001	1457.68 (1107.92–1917.84)	88.46 (64.22–121.86)	< 0.001	100.00	95.83	0.574
	II	273.86 (225.62–332.42)	228.21 (171.48–303.71)	0.276	68.47 (56.41–83.10)	16.22 (11.10–23.71)	< 0.001	100.00	89.36	0.014
	III	675.19 (561.49–811.92)	260.20 (178.35–379.61)	< 0.001	168.80 (140.37–202.98)	11.08 (6.12–20.05)	< 0.001	100.00	84.85	0.001
5	I	546.12 (408.61–729.91)	660.98 (549.64–794.86)	0.306	136.53 (102.15–182.48)	19.40 (14.51–25.95)	< 0.001	100.00	88.42	0.122
	II	209.42 (176.39–248.63)	122.10 (97.10–153.55)	< 0.001	52.35 (44.10–62.16)	7.62 (5.36–10.82)	< 0.001	100.00	78.26	< 0.001
	III	501.89 (417.41–603.47)	567.52 (397.01–811.27)	0.518	125.47 (104.35–150.87)	36.94 (24.12–56.56)	< 0.001	100.00	100.00	–

Group 1: low-dose Sabin IPV group. Group 2: medium-dose Sabin IPV group. Group 3: high-dose Sabin IPV group. Group 4: control Sabin IPV group. Group 5: control Salk IPV group

### Maternal antibody trichotomy

**Fig. 2:**
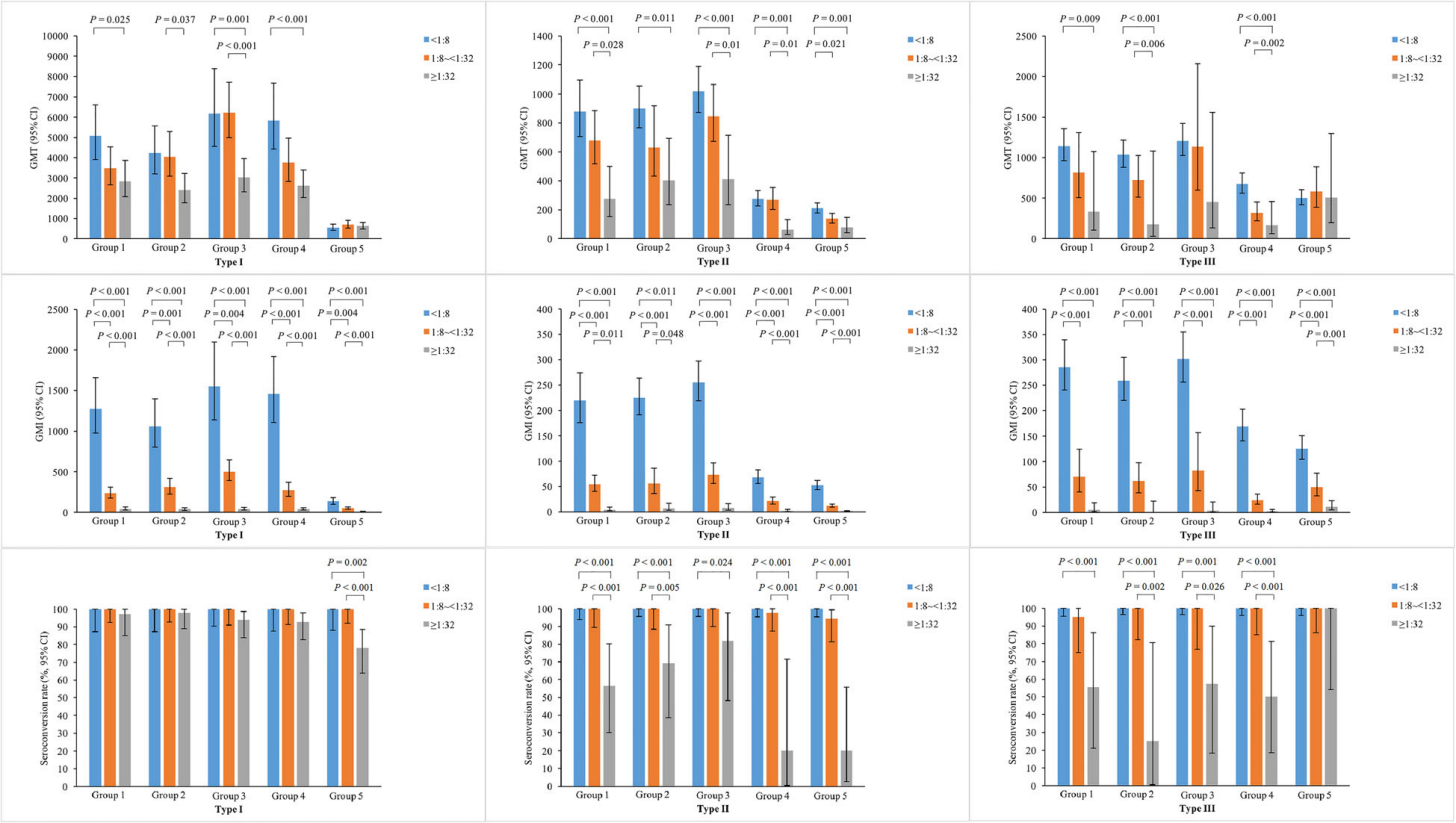
Multiple comparisons of GMTs, GMIs and seroconversion rates of poliovirus type-specific neutralizing antibodies after vaccination among participants with different maternal poliovirus antibody levels. Group 1: low-dose Sabin IPV group. Group 2: medium-dose Sabin IPV group. Group 3: high-dose Sabin IPV group. Group 4: control Sabin IPV group. Group 5: control Salk IPV group

Figure 2 shows the multiple comparisons of GMTs, GMIs and seroconversion rates of poliovirus type-specific neutralizing antibodies after vaccination among participants with different maternal poliovirus antibody levels. Among participants with maternal antibody titer < 1:8, 1:8 ~ < 1:32 and ≥ 1:32, the GMTs and GMIs of poliovirus antibodies after vaccination showed a tendency to decline with the increasing of maternal antibody levels. Significant differences in the GMTs after vaccination were found among participants with maternal antibody titer < 1:8, 1:8 ~ < 1:32 and ≥ 1:32 for type I poliovirus in low-dose Sabin IPV group, medium-dose Sabin IPV group, high-dose Sabin IPV group and control Sabin IPV group, for type II poliovirus in all five vaccine groups, for type III poliovirus in low-dose Sabin IPV group, medium-dose Sabin IPV group and control Sabin IPV group. Besides, the GMIs of three types of poliovirus antibodies in all five vaccine groups were significantly different among participants with different maternal poliovirus antibody levels. The seroconversion rates of three types of poliovirus antibodies in all five groups were comparable between participants with maternal antibody titer < 1:8 and 1:8 ~ < 1:32, ranged from 94.44 to 100%. While the seroconversion rates of antibodies in participants with maternal antibody titer ≥1:32 were significantly lower for type I poliovirus in control Salk IPV group, for type II poliovirus in all five vaccine groups, for type III poliovirus in low-dose Sabin IPV group, medium-dose Sabin IPV group, high-dose Sabin IPV group and control Sabin IPV group, ranged from 25.00 to 81.81%.

## Discussion

In our study, a certain weakening effect of maternal poliovirus antibodies on the immune responses of infants to poliovirus vaccines was observed, including the investigational Sabin IPV with different antigen contents, licensed Sabin IPV and Salk IPV. Among the five vaccine groups, the seropositive rates of maternal poliovirus type-specific antibodies were the highest for type I poliovirus, ranged from 70.87 to 78.40%, followed by those for type II poliovirus, ranged from 34.40 to 44.95%, and those for type III poliovirus were the lowest, ranged from 16.54 to 26.61%. The seropositive rates of maternal poliovirus type-specific antibodies in this study were similar to the results of a recent phase III IPV clinical trial [18]. This is because after years of OPV vaccination in China, high levels of poliovirus antibodies have formed in the population, resulting in high levels of maternal antibodies in newborns.

In the investigational Sabin IPV, control Sabin IPV and control Salk IPV groups, the effect of maternal poliovirus antibodies on poliovirus vaccines were consistent, this suggested that this effect may be a common problem in IPV. The results of maternal antibodies lowered the antibody responses of infants to IPV were also found in other randomized clinical trials [13–16], and this finding of our study is in accordance with that of a meta-analysis, where 2-fold higher maternal antibody concentrations resulted in 20 to 28% lower post-vaccination antibody concentration of IPV [12]. In another similar study conducted by us, the immune responses of IPV were attenuated by the high level of maternal poliovirus antibodies, but an opposite result showed that the post-vaccination GMTs for type I poliovirus were significantly higher among infants with high maternal antibody levels in Salk IPV group [19]. The exceptional result seemed to lack biological rationality, or it might be due to the small sample size and insufficient sample representativeness. Moreover, the negative effect of maternal poliovirus antibodies on the immune responses of poliovirus vaccines were found in low-dose Sabin IPV group, medium-dose Sabin IPV group and high-dose Sabin IPV group, which indicated that the increased antigen contents of polioviruses in vaccines may not diminish this effect of maternal poliovirus antibodies.

There were several limitations of this study. First, the blood samples for maternal poliovirus antibody detection were collected immediately before the first dose, when the infant participants were 2 months old, not the cord blood. But China has been certified as being polio free since 2000 [20], it is extremely unlikely for an infant to be infected with poliovirus within 2 months of birth. Thus, it is reasonable to consider pre-vaccination poliovirus antibodies as the maternal antibodies. Second, for type II and type III polioviruses, the proportion of participants with maternal antibody titer ≥1:32 was relatively small. This may have some influence on the results, and needs further study. Third, this study is a pooled analysis of three IPV clinical trials, not a randomized controlled study designed specifically to explore the effect of maternal poliovirus antibodies on the immune responses of infants to poliovirus vaccines.

Although the seroconversion rates of infants with high maternal antibodies are lower, the GMTs of them after immunization are still relatively high, which may protect them against polio. In addition, the presence of maternal antibodies can also provide protection for infants during their first few months of life. Considering the relatively poor immunogenicity of IPV in infants who were maternal antibody positive, especially in those with maternal antibody titer ≥1:32, an adjusted immunization strategy of IPV may be needed for them, such as delaying the vaccination. Besides, the effect of maternal poliovirus antibodies on the antibody persistence of IPV vaccination may be needed to be observed in long term, and we will take this as a potential focus of our subsequent work. In the future, maternal antibody will be an important factor to be considered in achieving precision immunization in infants [21].

## Conclusions

Overall, maternal poliovirus antibodies interfered with the immune responses of infants to poliovirus vaccines, and a high level of maternal antibodies exhibited a greater dampening effect.

## Data Availability

All authors had full access to the data and materials. Data are available within this article. Detailed data is available from the corresponding author upon reasonable request.

## Abbreviations

OPV: Oral poliovirus vaccine
IPV: Inactivated poliovirus vaccine
GMTs: Geometric mean titers
GMIs: Geometric mean antibody fold increases
